# Silent Dyspnea: Spontaneous Pneumomediastinum in a Heroin User

**DOI:** 10.7759/cureus.20496

**Published:** 2021-12-18

**Authors:** Saba Ali, Lanson B Colaco, Sreekrishnan Trikkur, Gireesh Kumar

**Affiliations:** 1 Department of Emergency Medicine, Amrita Institute of Medical Sciences, Kochi, IND; 2 Department of General Medicine, KVG Medical College & Hospital, Sullia, IND; 3 Department of Internal Medicine, JC Medical Center, Orlando, USA

**Keywords:** opioid epidemic, shortness of breath, unusal causes of persistent chest pain, inspiratory dyspnea, spontaneous pneumomediastinum (spm)

## Abstract

Spontaneous pneumomediastinum (SPM) is a relatively rare presentation that often follows a benign clinical course. It is mainly triggered by underlying bronchial asthma, respiratory tract infections, strenuous activities, or illicit drug use. We present a case of an isolated primary pneumomediastinum where the patient was a 24-year-old man with underlying bronchial asthma who presented with acute onset of shortness of breath and pleuritic chest pain following snorting of an opioid-heroin. Although the clinical exam and chest radiograph were both unremarkable, the multi-detector computed tomography of the chest revealed an isolated pneumomediastinum. The patient was managed conservatively in accordance with existing evidence as SPM is known for its spontaneous recovery.

## Introduction

Spontaneous pneumomediastinum (SPM) is the presence of gas in the mediastinum in the absence of trauma. It is rare unless it is accompanied by asthma or other lung pathology and potential predisposing triggers [1,2]. Hence, any literature that identifies and confirms SPM and its risk factors are of much importance. In the adult and pediatric populations, the reported incidence ranges from 1 in 800 to 1 in 42,000 people [3]. It is most common during the neonatal period, with a second peak in late infancy and early childhood [4]. The third peak occurs during adolescence, and it is most prominent in tall and thin males. The most common trigger is an acute asthma exacerbation, followed by a lower respiratory tract infection [1,5,6].

This case report discusses the clinical presentation, possible etiology, relevant investigations, and treatment plan of a young man who presented to our emergency department (ED) with isolated pneumomediastinum. Most importantly, the case presented here emphasizes the importance of considering SPM as a differential diagnosis in patients who present with acute chest discomfort or shortness of breath.

## Case presentation

A 24-year-old man presented to our emergency department (ED) with symptoms of four days of acute onset breathing difficulty, dry cough, and intermittent non-radiating chest pain of retrosternal origin and pleuritic character. A few hours following the onset of the symptoms, he also developed a low-grade fever. There was no history of vomiting, neck pain, lightheadedness, odynophagia, dysphagia, neck swelling, foreign body ingestion, hemoptysis, recent surgeries, trauma, or recurrent respiratory tract infections. Due to persistent symptoms, he sought treatment at a nearby hospital, where he was treated with cough suppressants, albuterol nebulization, and steroids. The chest radiograph (CXR) revealed normal parenchyma, whereas the high-resolution computed tomography chest (HRCT-chest) suggested an isolated pneumomediastinum. He was referred to our teaching hospital (quaternary care center) for further evaluation and treatment.

When he arrived at our department, the patient was conscious, oriented, had a patent airway, and spoke in complete sentences with no hoarseness of voice. He was tachypneic and had a modified Medical Research Council (mMRC) grade of 2 with peripheral oxygen saturation (SpO_2_) of 97% while receiving 2 L of oxygen through nasal prongs. He had a regular heart rate of 98 beats per minute and blood pressure of 120/80 mm Hg. All of his peripheral pulses were palpable. He had a fever and random fingerstick blood sugar of 164 mg/dL. Pupils were 2.5 mm bilaterally and symmetrically reactive to light. The arterial blood gas analysis, we used as our primary adjunct revealed no acid-base imbalance or hypoxemia. Serum lactate levels, as well as serum electrolytes, were normal. An electrocardiogram (ECG) was performed to rule out cardiac causes of chest pain, specifically pericarditis, and revealed sinus rhythm with no ST-T changes. A bedside lung ultrasound ruled out pneumothorax.

The patient smoked about two to three cigarettes every day for the past two years. He was diagnosed with bronchial asthma since childhood, for which he received ayurvedic (a form of complementary and alternative medicine) treatment. A detailed history revealed that he had inhaled an opiate, heroin, a day before his symptoms began.

**Figure 1:**
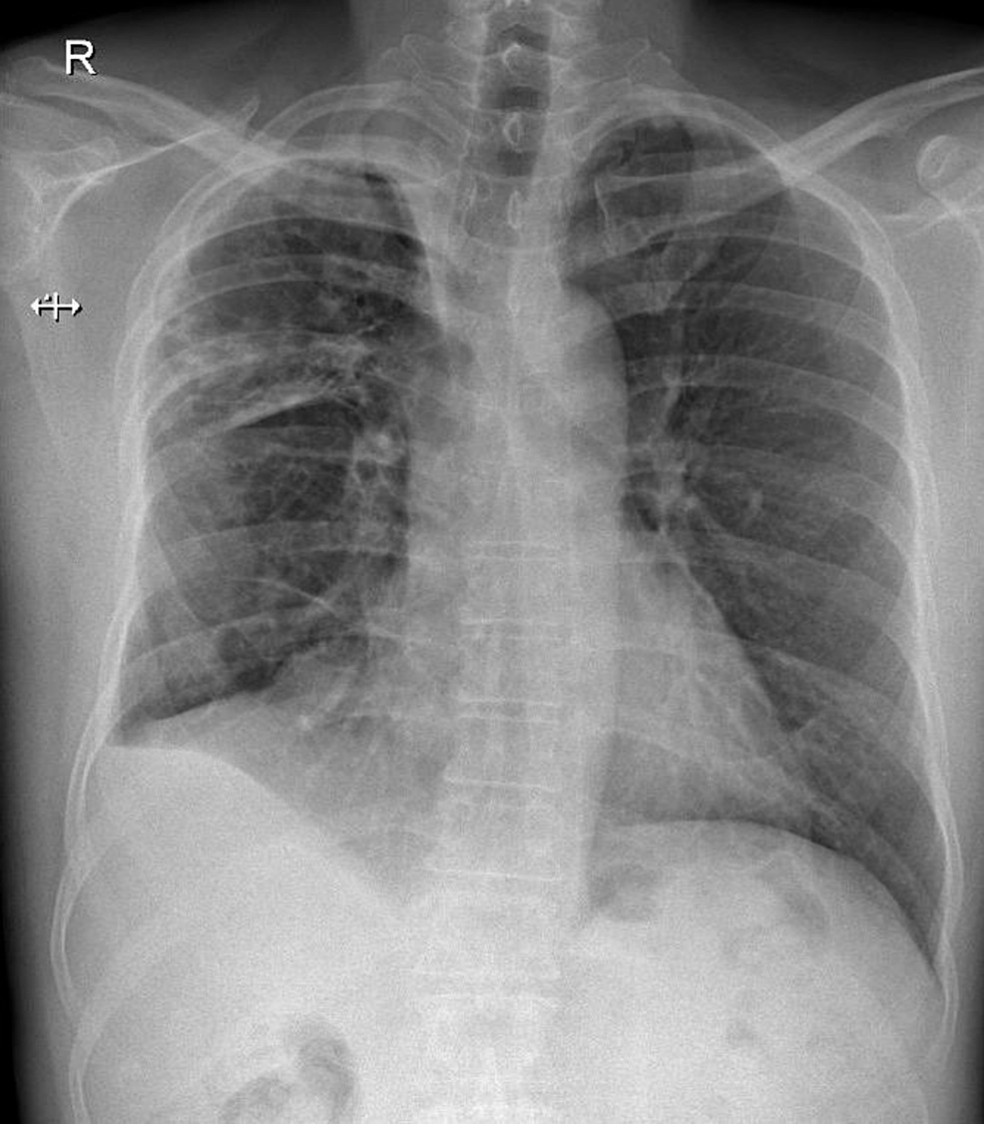
Chest radiograph (posteroanterior erect view) taken at presentation. No abnormal findings were noted.

**Figure 2:**
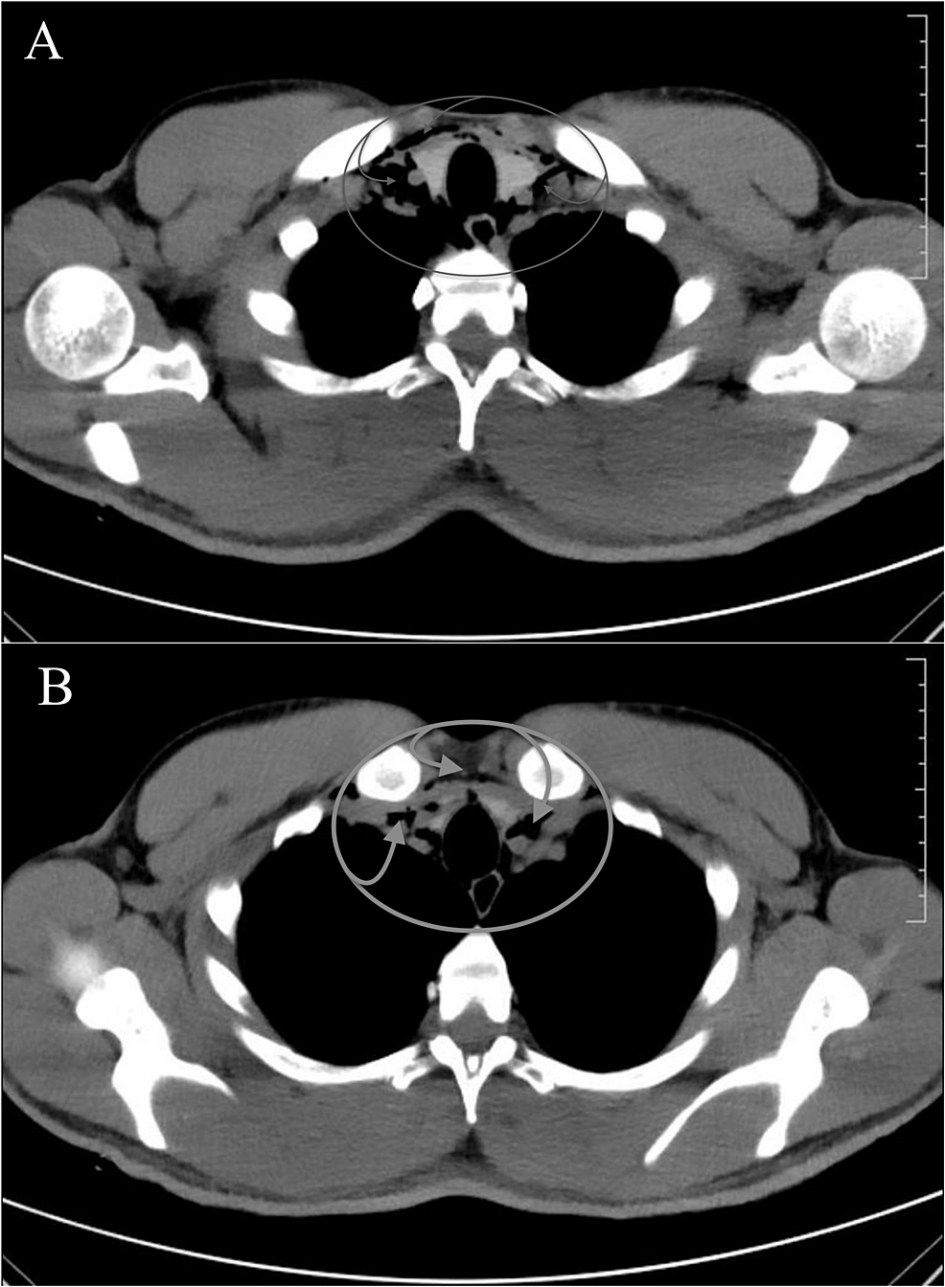
Multi-detector computed tomography scan of the patient’s chest demonstrating extensive pneumomediastinum and free air along the neck spaces, tracking to the retropharyngeal space and intermuscular plane of the neck, at slice 29 (A) and slice 32 (B).

He was tall and thin in stature, with a body mass index (BMI) of 19.8 kg/m^2^. His respiratory examination revealed bilateral equal air entry, symmetrical chest rise, and adequate chest exertion with a regular rhythm. The chest was clear on auscultation, with no additional sounds. There was no evidence of subcutaneous emphysema. The neck veins were not distended and the Hamman sign (crunching, rasping sound synchronous with the heartbeat heard over the precordium, primarily during systole and especially in lateral decubitus position, associated with muffling of heart sounds) was absent. The rest of his systemic examination was unyielding. The patient's other laboratory investigations were routine except for an elevated C-reactive protein level of 53.4 mg/L, a total leukocyte count of 14,000 cells/mm^3^, and a neutrophil differential of 80.2%. The CXR was normal (Figure 1), but a multi-detector computed tomography (MDCT) chest with contrast (repeated at our institute to investigate the progression of the pre-diagnosed pneumomediastinum) revealed extensive pneumomediastinum and free air along the neck spaces, tracking to the retropharyngeal space and intermuscular plane of the neck, with no evidence of hollow viscus perforation or definite communication between the trachea and the esophagus (Figure 2). There was no evidence of pneumothorax or pneumopericardium.

As a result, the patient was diagnosed with spontaneous, uncomplicated pneumomediastinum, with underlying asthma and recent opioid use as potential triggers, which may have caused sustained physical exertion. He was treated conservatively with adequate rest and analgesia, oral antibiotics, and oxygen support as needed. Valsalva and forced expiration maneuvers and spirometry were avoided, while his underlying asthma was treated. The patient's admission was otherwise uneventful, and he was discharged after a full recovery in around 24-48 hours.

## Discussion

Spontaneous pneumomediastinum (SPM) is a rare condition that follows a benign clinical course [7,8]. It is defined as the presence of gas within the mediastinum with no clearly identifiable precipitating factor. If spontaneous pneumomediastinum develops, it is usually associated with underlying bronchial asthma, recent physically demanding activities, or inhalation of illicit drugs [1,9,10]. It is predominantly observed in young men with a tall thin body physique [7,11].

Pneumomediastinum is categorized into primary (SPM) and secondary types. SPM usually has an underlying lung pathology, such as cystic fibrosis, respiratory tract infection, and asthma or precedes a prolonged strenuous activity such as parturition, inhalational/intravenous illicit drug administration, weight-lifting, or emesis. On the other hand, secondary pneumomediastinum occurs secondary to blunt or penetrating thoracic trauma [2,12]. The incidence rate of SPM among children and adolescents presenting for emergency treatment of asthma is between 0.3 and 5% [3,4]. According to one study, when young people presenting to an ED with acute chest pain or dyspnea were routinely screened with CXR, many cases of SPM were discovered [11].

The pathogenesis of SPM involves air leaking into the surrounding bronchovascular sheath through the rupture of alveoli [11]. Since the mean pressure in the mediastinum is always more negative than the pulmonary parenchymal pressure, air flows centripetally along the vascular sheaths, most likely aided by the pumping action of breathing. It spreads through the loose mediastinal fascia to the thoracic subcutaneous tissues (pneumopericardium and pneumothorax), upper limbs, and neck, rarely reaching the spine (pneumorrhachis) [13-15]. Patients who smoke marijuana or use heroin (as in our case) are observed to take deep breaths and hold them to perform a Valsalva maneuver during the drug administration process. We hypothesize this to be the trigger for SPM in our patients [12].

In decreasing order of frequency, the most common clinical presentations are chest pain, shortness of breath, cough, neck pain, odynophagia, or dysphagia [5,16]. Among the many other causes of chest pain, such as acute coronary syndrome and pericarditis, the primary differential diagnoses to consider are esophageal perforation, pneumothorax, and pulmonary embolism [17]. Complications can occur, including tension pneumomediastinum, tension pneumothorax, and increased pulmonary pressure worsening respiratory distress. However, most cases undergo spontaneous resolution [18].

A plain CXR is diagnostic in only up to up to 70% of cases [19]. If the CXR fails to show an SPM, especially in patients with symptoms like dysphagia, a high index of clinical suspicion should be exercised, and an MDCT-chest is recommended. Management is conservative and requires only 24-48 hour monitoring for complications (such as ruptured viscus) [17]. Despite the fact that antibiotics were administered in this case, there is no evidence to support their usage in this setting [20].

## Conclusions

Spontaneous pneumomediastinum is usually a benign condition that frequently goes undiagnosed and responds very well to conservative treatment. It should be considered a differential diagnosis when any healthy adolescent or young adult presents with acute chest pain or shortness of breath. SPM should especially be ruled out in people who have recently used illegal drugs and experience acute chest pain and dyspnea. Although most SPM cases resolve spontaneously, there is a chance that it may progress to a fatal pneumothorax, so clinicians must be aware of this entity and plan patient care accordingly.
